# Seasonality of healthcare-associated *Stenotrophomonas maltophilia*

**DOI:** 10.1017/ice.2022.280

**Published:** 2023-09

**Authors:** Dianne B. Auld, Phinnara Has, Leonard A. Mermel

**Affiliations:** 1 Department of Epidemiology and Infection Control, Lifespan Hospital System, Providence, Rhode Island; 2 Biostatistics, Epidemiology and Research Design, Lifespan Hospital System, Providence, Rhode Island; 3 Division of Infectious Diseases, Department of Medicine, Warren Alpert Medical School of Brown University, Providence, Rhode Island

## Abstract

From April 1, 2016, through March 31, 2022, growth of *Stenotrophomonas maltophilia* from clinical specimens at our academic medical center was significantly more likely during July–September than during other calendar quarters.

Gram-negative bacterial infections peak during summer months^1,2^ and a predominance of gram-negative infections is associated with proximity to the equator.^3^ A Belgian national cohort study of hospital-associated bloodstream infections reported that infections due to *Stenotrophomonas maltophilia* peaked during summer months.^2^ Additionally, hospital-associated bloodstream infections due to *S. maltophilia* demonstrated the greatest seasonal variability compared with other bacteria. Based on these findings, we hypothesized that we would find a summer peak in isolation of *Stenotrophomonas maltophilia* from clinical specimens in our hospital.

## Methods

This retrospective study included *S. maltophilia* isolated from clinical specimens at our tertiary-care, level 1 trauma center from April 1, 2016, through March 31, 2022. We defined *S. maltophilia* isolates as healthcare-associated if a clinical specimen was obtained from a patient >48 hours after hospital admission or within 48 hours of hospitalization if a patient had been previously admitted to our hospital over the prior 30 days. Only the first isolate was included in our analysis if a patient had >1 clinical specimens that grew *S. maltophilia*. Statistical methods included Poisson regressions to model the counts of quarterly hospital-acquired infections of *S. maltophilia*. All tests were 2-sided; *P* < .05 was considered statistically significant.

## Results

**Fig. 1.**
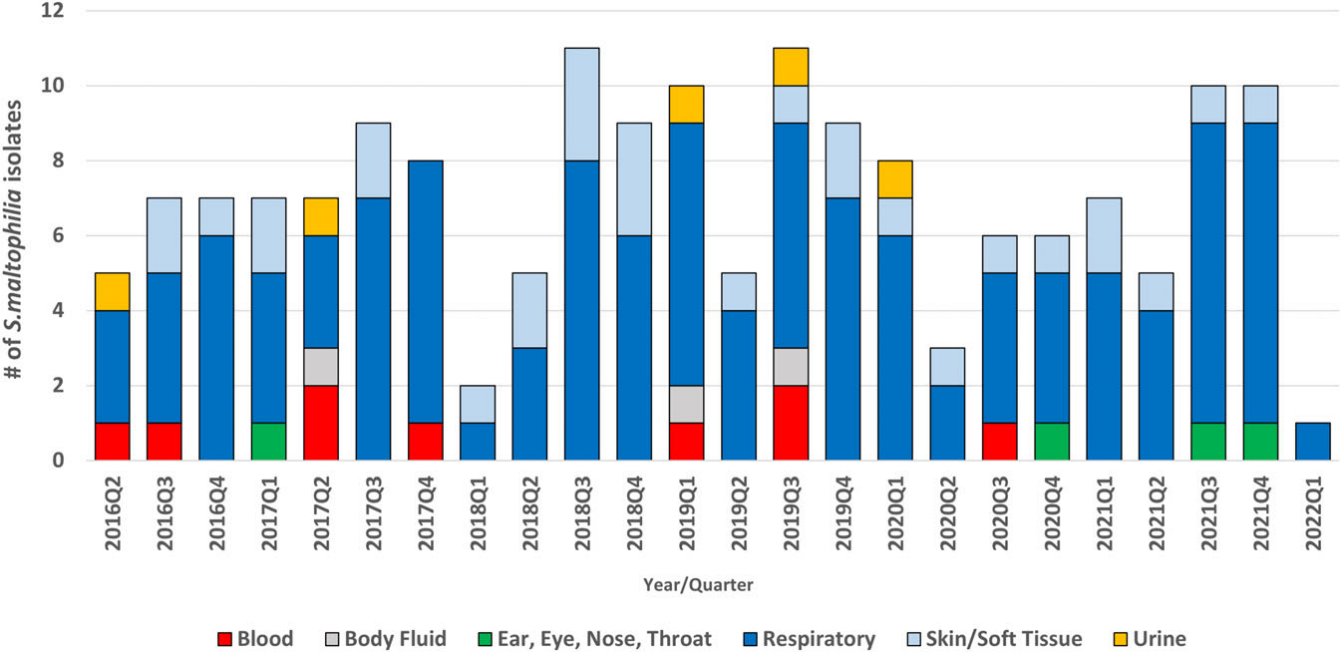
Healthcare-associated *Stenotrophomonas maltophilia* clinical isolates, April 1, 2016–March 31, 2022.

Respiratory specimens accounted for 53 (69%) of 172 *S. maltophilia* isolates (Fig. 1). We detected a significant increase in *S. maltophilia* isolates from July through December (calendar quarters 3 and 4) compared to January through June (calendar quarters 1 and 2; relative risk [RR], 1.85; 95% confidence interval [CI], 1.10–2.02; *P* < .001). The greatest peak was identified July through September: quarter 3 vs quarter 1: RR, 1.54 (95% CI, 1.01–2.36; *P* = .046); quarter 3 vs quarter 2: RR, 1.80 (95% CI, 1.15–2.81; *P* = .01); quarter 3 vs quarter 4: RR, 1.10 (95% CI, 0.75–1.62; *P* = .62). Otherwise, quarter 4 had a significantly higher peak than quarter 2 (RR, 1.63; 95% CI, 1.04–2.57; *P* = .03).

## Discussion

We have demonstrated seasonality of *S. maltophilia* among clinical isolates at our medical center. We can only speculate as to why this is the case. The concentration of bacteria in potable water peaks during summer months, and the greatest degradation of water quality occurs after overnight stagnation of potable water during the summer.^4^ Additionally, a peak in the concentration of coliform bacteria in streams, rivers, and drinking wells has been documented during the summer months.^5^

Nonfermenting gram-negative bacteria such as *S. maltophilia* have been isolated from drinking water, and hospital outbreaks of *S. maltophilia* infections have been traced to tap water dispensed from faucets with aerators.^6^ It is unclear whether nonfermenting gram-negative bacteria such as *S. maltophilia* have a selection advantage based on the temperature, pH, and oxygen content that might be found in water sources during summer months.^7^ Our hospital has a continuous feed of monochloramine into our potable hot water in patient-care buildings to reduce the risk of hospital-acquired *Legionella* infections. Although a nonsignificant increase in the number of faucets and showerheads growing *S. maltophilia* ^8^ and other nonfermenting gram-negative bacteria^9^ has been documented in hospitals with monochloramine potable water treatment, we were unable to identify any hospital outbreaks due to *S. maltophilia* associated with monochloramine water treatment. Various infection prevention strategies may be used to reduce risk of nosocomial infections due to *S. maltophilia* and other nonfermenting gram-negative bacteria. These include minimizing tap water use, removing aerators from faucets, use of splash guards around sinks, and placement of point-of-use filters in high-risk areas among other interventions.^10^

Our study had several limitations. We included clinical isolates without medical record review, so we cannot be certain whether the isolates represented true infections, colonization, or possible contamination. Additionally, we were limited by a relatively small number of clinical isolates. The study was conducted at a single center, so these results may not be generalizable to other populations or settings. We did not do widespread testing of our hospital potable water to assess for the presence of *S. maltophilia*, which may have been epidemiologically linked to some of the clinical isolates.

In conclusion, we confirmed the seasonality of *S. maltophilia* isolated from clinical isolates with a significant peak observed from July through September. This finding may be due to changes in potable water composition during the summer months. Further research is needed to better understand the determinants driving seasonality of *S. maltophilia* and other nonfermenting gram-negative bacteria that may cause infections in healthcare settings. Additionally, the data presented herein, along with similar publications, should prompt discussion regarding potential seasonal adjustment for surveillance programs involving healthcare-associated infections caused by *S. maltophilia* and other pathogens that clearly display seasonality.
